# Is the processing of affective prosody influenced by spatial attention? an ERP study

**DOI:** 10.1186/1471-2202-14-14

**Published:** 2013-01-29

**Authors:** Julia C Gädeke, Julia Föcker, Brigitte Röder

**Affiliations:** 1 Biological Psychology and Neuropsychology, University of Hamburg, Von-Melle-Park 11, Hamburg, 20146, Germany; 2 Department of Psychology and Educational Sciences, University of Geneva, Boulevard du Pont-d'Arve 40, Geneva, 1205, Switzerland

**Keywords:** Voices, Prosody, Spatial attention, Emotion, ERPs

## Abstract

**Background:**

The present study asked whether the processing of affective prosody is modulated by spatial attention. Pseudo-words with a neutral, happy, threatening, and fearful prosody were presented at two spatial positions. Participants attended to one position in order to detect infrequent targets. Emotional prosody was task irrelevant. The electro-encephalogram (EEG) was recorded to assess processing differences as a function of spatial attention and emotional valence.

**Results:**

Event-related potentials (ERPs) differed as a function of emotional prosody both when attended and when unattended. While emotional prosody effects interacted with effects of spatial attention at early processing levels (< 200 ms), these effects were additive at later processing stages (> 200 ms).

**Conclusions:**

Emotional prosody, therefore, seems to be partially processed outside the focus of spatial attention. Whereas at early sensory processing stages spatial attention modulates the degree of emotional voice processing as a function of emotional valence, emotional prosody is processed outside of the focus of spatial attention at later processing stages.

## Background

Vocal prosody is one of the most important features of human communication enabling individuals to recognize the affective state of people in order to react quickly and appropriately in social situations. Changes in respiration, phonation, and articulation determine the acoustic signal of a voice and accompany emotional reactions, similar as changes in facial expressions [1].

The encoding of emotional prosody is based on various acoustic features, such as pitch and pitch variation, syllable duration, and voice quality [2]. The quality of the voice is determined by different laryngeal and supralaryngeal aspects. The extraction of emotional prosody has been suggested to be automatic [3-5]. Event-related potential studies have shown that similar to the processing of facial expression, emotional vocalizations (such as screams) elicit a fronto-central positivity 150 ms after stimulus onset compared to frequency-matched artificial sounds [6]. Other studies have focused on the dissociation between vocal emotional processing and the processing of person-identity information in human voices [2]. Early ERP priming effects have been observed for happy voices but not for sad voices in the time range of the P2, a positivity with a latency of 200 ms, whereas the ERP speaker identity matching effect did not start until around 300 ms [2]. The authors referred their results to physical differences related to emotional prosody. They suggested that higher frequencies are presented in happy voices than in sad voices, which might contribute to a faster and more efficient processing of happy vocal stimuli [2]. Another study has shown a modulation of ERPs by the emotional valence of voices in the P200 time range as well [7]. The authors interpreted their results as evidence for a rapid emotional decoding.

In humans, an enhanced brain activity has been observed to emotional compared to neutral voices in auditory association areas including the superior temporal sulcus (STS) [3,8-10] and the right middle superior temporal gyrus [11-13]. These activation patterns have been observed irrespectively of which vocal prosody was used [3,4,9]. A recent study used functional near-infrared-spectroscopy (fNIRS) and reported an increased activation in the auditory cortex in response to pleasant and unpleasant sounds in comparison to neutral sounds [14]. This activation pattern suggests that even sensory areas differentially respond to emotional prosodies, which nicely matches corresponding results in the visual system [5]. However, brain imaging data do not allow to conclude whether changes of sensory cortex activity are due to changes in bottom up processing or due to feedback connections of higher cortical areas or the amygdala.

It has been a matter of debate whether the processing of emotional information depends on attentional resources [15]. There are a number of studies suggesting that attention is necessary to select basic stimulus features in order to store relevant stimulus properties in working memory [16]. However, there is evidence as well suggesting that emotional signals can be processed independent of attention and awareness and may guide attention to salient stimuli [17].

Although there are a few studies suggesting that the processing of facial expression requires attention [15,18,19] many of the recent studies are compatible with the view that emotional features can be processed automatically. For example, a processing of emotional faces has been observed outside the focus of attention [20] and even in the disregarded space by neglect patients [21,22]. By contrast, additional studies have shown that attention is capable to further enhance the processing of emotional facial expressions [17,23]. These results suggest for visual emotional stimuli both some attention independent processing but also some top down control. This combination seems highly efficient since many emotional stimuli in the environment are totally irrelevant for an individual. Thus, an individual must be able to inhibit an orienting to emotional stimuli in order not to interfere with current action goals.

Interestingly, brain networks involved in emotional processing show an overlap with attentional networks: Both attentional and emotional processes have been found to activate higher cortical areas such as parietal, frontal and cingulated areas as well as subcortical regions [17 for an overview]. However, the emotion specific activation of the amygdala might allow emotional information to be processed prior to the attentive stimulus selection [17] thus enabling emotional features to serve as exogenous cues that guide attention to relevant events.

While interactions between attention and emotional processing in the visual modality have been extensively studied, the question whether the processing of emotional prosody depends on attention or not has been addressed only recently. The situation for emotional voices might be quite different than for emotional faces, since the latter requires an orientation of the eyes toward visual stimuli in order to perceive them with a sufficient accuracy, while such an overt orienting response is not necessary in order to process emotional voices.

Sander and Scheich [24] found amygdala activity in response to affective non-verbal vocalizations (laughing and crying) regardless of whether the participants attended to the emotional valence of the stimuli or were engaged in a distracter task. Moreover, Grandjean et al. [3] showed that emotion-related activity in response to angry voices in the middle right STS did not vary with selective spatial attention, suggesting a preattentive processing of emotional prosody. Using the same paradigm, Sander et al. [4] replicated these findings and extended them to the amygdala. Additionally, as for visual neglect [21,22], auditory extinction was found to be attenuated for stimuli with an emotional as compared to a neutral prosody [25]. More recent studies, however, challenge the assumption of a total automaticity of emotional prosody processing. Mothes-Lasch et al. [26] presented voices of seven different emotional prosodies: Participants had to classify the gender of the speaker or they had to perform a difficult visual discrimination task. The authors found a higher response of the amygdala to angry compared to neutral voices only in the auditory but not in the visual task suggesting that orienting attention away from the auditory modality (intermodal attention) abolishes emotional prosody processing. By contrast, emotional voice can serve as an exogenous crossmodal attention cue. Brosch et al. [27] showed that reaction times in a dot probe task were shorter when the visual target was presented on the side at which an angry utterance (compared to a neutral utterance) was heard just before. The parallel recorded ERPs revealed an enhanced amplitude of the visual P1 to the target when an emotional voice was presented at the same side as the visual target compared to when the visual target was presented at the side of the preceding neutral voice [28].

Our study extends previous work by investigating the time course of spatial attention effects under different emotional prosody conditions. Thus, the present study complements the findings of imaging studies using a dichotic listening paradigm in order to analyze spatial attention effects on the processing of human angry and neutral voices [3].

The present ERP-study orthogonally manipulated the focus of spatial attention and the emotional prosody of the stimuli in order to analyze whether or not physically identical emotional stimuli are processed differently within and outside the focus of spatial attention. Pseudo-words comprising two identical (standards) or two different (deviants) syllables spoken by two different female voices were presented randomly on the left and on the right side of the participant. In different blocks, participants had to attend to one of the two spatial positions. They had to respond to infrequent (p=0.05) deviant stimuli presented at the attended position. The present experiment, therefore allowed for a direct comparison of the processing of physically identical emotional stimuli once when spatially attended and once when unattended.

Based on previous findings, we expected spatial attention to enhance ERPs to vocal stimuli starting around 100 ms after stimulus onset (i.e., the auditory N1).

If emotional prosody is processed in the absence of spatial attention, ERP modulations due to emotional valence are expected to be independent of the focus of spatial attention and thus additive to the ERP spatial attention effects. By contrast, if attention is necessary to process emotional valence, an effect of emotional valence is predicted only for the spatially attended stimuli or is different for spatially attended and unattended stimuli.

## Methods

### Participants

Seventeen healthy young student participants took part in the main experiment. According to self-report all participants had normal hearing and normal or corrected to normal vision. They were either paid for participation or received course credits. The experiment was conducted in accordance with the ethical guidelines laid down in the Declaration of Helsinki (2000). Because of low performance (see procedure), only data of thirteen participants (20 to 28 years, mean age 23 years, 7 females) were analyzed.

### Stimuli

The final stimulus set consisted of nine different two-syllable pseudo-words (pronounceable German non-words) spoken by two actresses in four different emotional prosodies (neutral, happy, threatening and fearful), resulting in 72 physically different stimuli. Three of the pseudo-words were deviant stimuli (two different syllables); the remaining six were standards (two identical syllables).

### Stimulus selection and evaluation

In a first step 48 two-syllable pseudo-words comprising two identical syllables (e.g. fefe, gigi), and 20 pseudo-words with two different syllables (the second consonant or vowel differed from the first; e.g. fefi, giki) were generated. Stimuli with two identical syllables were later used as standards, those with two different syllables as deviants or targets. Two actresses spoke these pseudo-words three times in four different emotional prosodies (happy, fearful, threatening, and neutral). Stimuli were recorded with a DAT recorder in an anechoic chamber. They were transferred to the computer and saved as wav-files. Preprocessing was done with the GOLDWAVE software (http://www.goldwave.com). The volume of the single sound files was equalized by setting the root mean square of each stimulus to 0.025.

The best two of the three recordings of each pseudo-word and each speaker were preselected by one of the authors (JG) for an evaluation study in which 24 students of the University of Marburg (19 to 34 years, mean 23 years, 22 females) rated each of the remaining 1088 stimuli (68 pseudo-words*2 voices* 2 versions* 4 emotions) on three dimensions (valence, dominance, arousal) using scales from −3 to +3 (valence: unpleasant (−3) – pleasant (3); dominance: submissive (−3) – dominant (+3); arousal: calming (−3) – stimulating (+3)).

The nine experimental stimuli (six standards and three deviants in the four emotional expressions spoken by the two voices) were selected using the criteria of duration as well as ratings of valence. Stimuli shorter than 250 ms or longer than 1020 ms were discarded. Rating values were transformed on a scale ranging from 1 (equivalent to −3) to 7 (equivalent to +3). In order to make sure that stimuli of different emotional categories would differ in their perceived valence, cutoff scores for the mean valence ratings were applied. These cutoff scores (neutral: 3.5 and 5.5; happy: 5 and 7; threatening: 1 and 2.5; fearful: 2 and 3.5) were defined to guarantee distinct stimuli for each voice in the four emotional categories.

For different sets of stimuli, analyses of variance with the factor Emotional Prosody (four levels) were calculated for the following dependent variables: Duration, Pitch, Intensity, Valence Rating, Dominance Rating, and Arousal Rating. For the final set of standard stimuli, the duration did not differ between emotion conditions (*F*(3,15) = 1.80, *p* > .1). Mean pitch differed between emotions (*F*(3,15) = 43.48, *p* < .001). The mean pitch of neutral vocal stimuli was significantly lower compared to happy, threatening and fearful voices (*p*s < .01 (neutral versus happy: *t*(5) = −8.53, *p* < .001; neutral versus threatening: *t*(5) = −11.63, *p* < .001; neutral versus fearful: *t*(5) = −7.40, *p* < .01). Moreover, happy voices had a higher pitch compared to threatening and fearful voices (*p*s < .05) (happy versus threatening; *t*(5) = 3.44, *p* < .05; happy versus fearful: *t*(5) = 4.99; *p* < .01). Threatening and fearful voices did not differ in the mean fundamental frequency (threatening versus fearful: *t*(5) = 0.392, *p* > .1).

Valence ratings (standard stimuli) depended on Emotional Prosody (*F*(3,15) = 308.76, *p* < .001). All pairwise comparisons between emotional prosodies were significant (all *p*s < .01). Dominance ratings depended on Emotional Prosody as well (*F*(3,15) = 667.27, *p* < .001). All differences between emotional prosodies in dominance ratings were significant (all *p*s < .01). By contrast, arousal ratings for the four emotional prosodies did not differ (*F*(3,15) = .15, *p >* .1).

Table 1 lists the means and standard errors of duration (ms), pitch (Hz), intensity (dB) and valence rating, dominance rating, and arousal rating separately for the four emotional prosodies for the final set of standard stimuli and Table 2 the same values for deviant stimuli.

**Table 1:** Item statistics: Mean (M) and Standard error of the mean (SE) of duration, pitch, intensity, valence ratings, dominance ratings and arousal ratings of standard stimuli in the different emotional prosodies spoken by the two different voices

**Emotional prosody**	**Duration (ms)**		**Pitch (Hz)**		Intensity * (dB)	
	**M**	**SE**	**M**	**SE**	**M**	**SE**
neutral	632	35	176	3.78	62.31	0.01
happy	575	57	271	17.45	62.25	0.001
threatening	602	56	244	8.78	62.15	0.19
fearful	518	44	252	11.24	62.25	0.02
**Emotional Prosody**	**Valence rating (1–7)**		**Dominance rating (1–7)**		**Arousal rating (1–7)**	
	**M**	**SE**	**M**	**SE**	**M**	**SE**
neutral	4.77	0.27	4.29	0.13	4.59	0.43
happy	5.37	0.08	4.56	0.12	4.84	0.32
threatening	1.92	0.13	6.30	0.07	4.81	0.38
fearful	2.76	0.14	2.23	0.14	4.63	0.21

*Intensity refers to the degree of *energy* which is present in a given sound wave. It is directly proportional to amplitude, or, more precisely, it is directly proportional to the squared amplitude (definition Praat, Help menu, http://www.fon.hum.uva.nl/praat/).

**Table 2:** Item statistics: Mean (M) and Standard error of the mean (SE) of duration, pitch, intensity, valence ratings, dominance ratings and arousal ratings of deviant stimuli in the different emotional prosodies spoken by the two different voices

**Emotional Prosody**	**Duration (ms)**		**Pitch (Hz)**		Intensity * (dB)	
	**M**	**SE**	**M**	**SE**	**M**	**SE**
neutral	731	60	186	2.69	62.21	0.01
happy	490	42	363	14.57	62.36	0.01
threatening	721	96	297	10.14	62.30	0.03
fearful	426	44	312	14	62.51	0.06
**Emotional Prosody**	**Valence rating (1–7)**		**Dominance rating (1–7)**		**Arousal rating (1–7)**	
	**M**	**SE**	**M**	**SE**	**M**	**SE**
neutral	4.83	0.51	4.50	0.15	3.98	0.50
happy	5.17	0.09	4.43	0.17	5.06	0.43
threatening	1.74	0.15	6.51	0.10	5.53	0.10
fearful	2.60	0.14	1.97	0.12	4.56	0.18

*Intensity refers to the degree of *energy* which is present in a given sound wave. It is directly proportional to amplitude, or, more precisely, it is directly proportional to the squared amplitude (definition Praat, Help menu, http://www.fon.hum.uva.nl/praat/).

The difference in duration of the deviant stimuli in different emotional prosodies was not significant (*F*(3,6) = 7.661 *p* < .1). Dominance ratings depended on Emotional Prosody (*F*(3,6) = 409.98, *p* < .001). All pairwise comparisons were significant (all *p*s < .01), with the exception of the comparison neutral vs. happy (*p* > .1). By contrast, arousal ratings did not depend on Emotional Prosody (*F*(3,6) = .15, *p* > .1).

The final stimulus set consisted of nine different two-syllable pseudo-words (pronounceable German non-words) each spoken by the two actresses in four emotional prosodies, resulting in 72 physically different stimuli. Three of the pseudo-words were deviant stimuli (two different syllables), the remaining six were standards (two identical syllables).

### Procedure

#### Training

In order to learn to discriminate the voices and to get familiar with the experimental procedure all participants took part in a 3–3.5 hours training session, one or two days prior to the EEG session. The training session consisted of five different units each of which had to be performed three times. The stimuli (six standards and two deviants in the four emotional expressions) were different from those used for the main experiment but came from the same stimulus pool. Since we did not analyze the factor voice^a^, the first four training units are not described in detail. The last phase of the training session was identical to an experimental block.

### Main experiment

For the main experiment the stimuli were presented from two speakers positioned in front of the participant at a distance of 1.4 m, one 45° to the left and one 45° to the right of the participant. Stimuli of both voices and of all emotional prosodies were presented with an equal probability and in a random order from the left and right speaker. Stimulus onset asynchronies varied between 1300 ms to 1700 ms with a mean of 1500 ms.

Participants were instructed to attend to stimuli which were presented at one of the two spatial positions (left or right) and which were spoken by one of the two female speakers. Their task was to respond by lifting the left or right index finger out of a light gate whenever they detected one of the deviant stimuli spoken by the attended voice and presented at the attended position (i.e. targets). Response hand was switched after half of the trials (from left to right index finger or vice versa). A specific instruction concerning the varying affective prosodies was not given. Thus, there were four experimental conditions (attend voice I vs. attend voice II and attend left vs. attend right speaker). Only the spatial attention effects were analyzed^a^.

The experiment comprised 16 blocks lasting for six to seven minutes each (four blocks for each of the four experimental conditions). A block comprised 192 standard stimuli (80%) and 48 deviant stimuli (20%), 24 of which were targets (5%). Attention instruction was changed every two blocks. Participants were blindfolded throughout the experiment. The correct position of the cap was achieved by aligning it at the nasion, the inion, and the preauricular points. The participant’s head was immobilized by using a chin rest. Moreover, participants were instructed to avoid excessive blinking, eye and head movements during a run. Breaks after a block were allowed whenever the participant wanted. The duration of the EEG experiment without any breaks was about 1.5 hour. Including breaks, practice and the electrode-preparation and removal, the whole experimental session lasted between 5 and 6 hours.

### Electroencephalographic recording

The EEG was continuously recorded from 61 Ag/AgCl electrodes mounted equidistantly in an elastic cap (Falk Minow Services, Munich). The central electrode M_4 (see data analyses) is positioned between Fz and Cz of the international 10–20 system. The horizontal electro-occulogram (HEOG) was assessed with a bipolar recording of two electrodes attached to the outer canthi of the eyes, the vertical EOG (VEOG) was monitored with an electrode placed under the right eye against the common reference. All electrodes were referenced to the right earlobe during recording and were re-referenced off-line to the averaged left and right earlobe references. Impedances were kept below 5 kΩ for scalp recordings and below 10 kΩ for EOG recordings by preparing the skin of participants with Every (Meditec SRI, Negernbotel) and alcohol. ECI Electrogel (Electrocap International, Ohio, USA) served as the electrolyte for all electrodes. The ground electrode was placed on a position at the middle of the forehead (below Fpz). Signals were amplified with two SynAmps-amplifiers (NeuroScan, Inc. Sterling, USA). The sample rate was 500 Hz and the bandpass was set to 0.1 – 100 Hz. Signals were recorded continuously and saved on a hard disc.

### Data analyses

#### Behavioral data

A response was classified as a hit if it occurred within a time window of 200 to 1700 ms following a target stimulus. All other responses were considered as false alarms (FA). Trials in which the participants did not respond to a target were defined as misses. The miss rate was derived by dividing the total number of misses by the total number of target trials. Correct rejections were defined as non-responses to deviants at the non-attended location.

Performance accuracy for discriminating the positions as a function of emotional prosody was assessed for each participant by calculating d’ (*d* ’ = *z*(*p*(*hit*)) − *z*(*p*(*FA*)); [29]). The hit rate was defined as the number of correct responses to target stimuli spoken by the attended voice at the attended position divided by the total number of targets. The false alarm rate (FA rate) was defined as the number of incorrect responses to deviant stimuli spoken by the attended or by the unattended voice but at the unattended position divided by the number of deviants at the unattended position.

Mean reaction times (RT) and percent correct were calculated for each condition and participant from which we derived inverse efficiency scores) (IE) in order to compensate for possible speed-accuracy trade offs ([30], see below). For calculating IE scores, mean RT are divided by percent correct [31]. Trials with reaction times below 200 ms or exceeding 1700 ms were disregarded. Analyses of Variance (ANOVAs) with the repeated measurement factor Emotional Prosody (four levels: neutral, happy, threatening, and fearful) were run for the dependent variables d’ and inverse efficiency scores. A main effect of Emotion was further analysed with t-tests (two-tailed) for dependent samples. Percent correct (%) and reaction times are reported in Table 3.

**Table 3:** Mean reaction times (ms) and mean accuracy (%) for each emotional prosody (neutral, happy, threatening, fearful) with standard errors of the mean

	**Neutral**		**Happy**		**Threatening**		**Fearful**	
	**mean**	**SE**	**mean**	**SE**	**mean**	**SE**	**mean**	**SE**
Percent correct (%)	76	4	51	2	53	3	60	3
Reaction Times (ms)	1084	24	925	35	1092	24	950	25

### ERP data

The continuous EEG was epoched from 100 ms prior to the stimulus onset until 1000 ms after stimulus presentation separately for each participant and condition. The pre-stimulus interval was defined as baseline. Only segments following standard stimuli were analyzed, segments with responses to standard stimuli were discarded.

**Figure 1:**
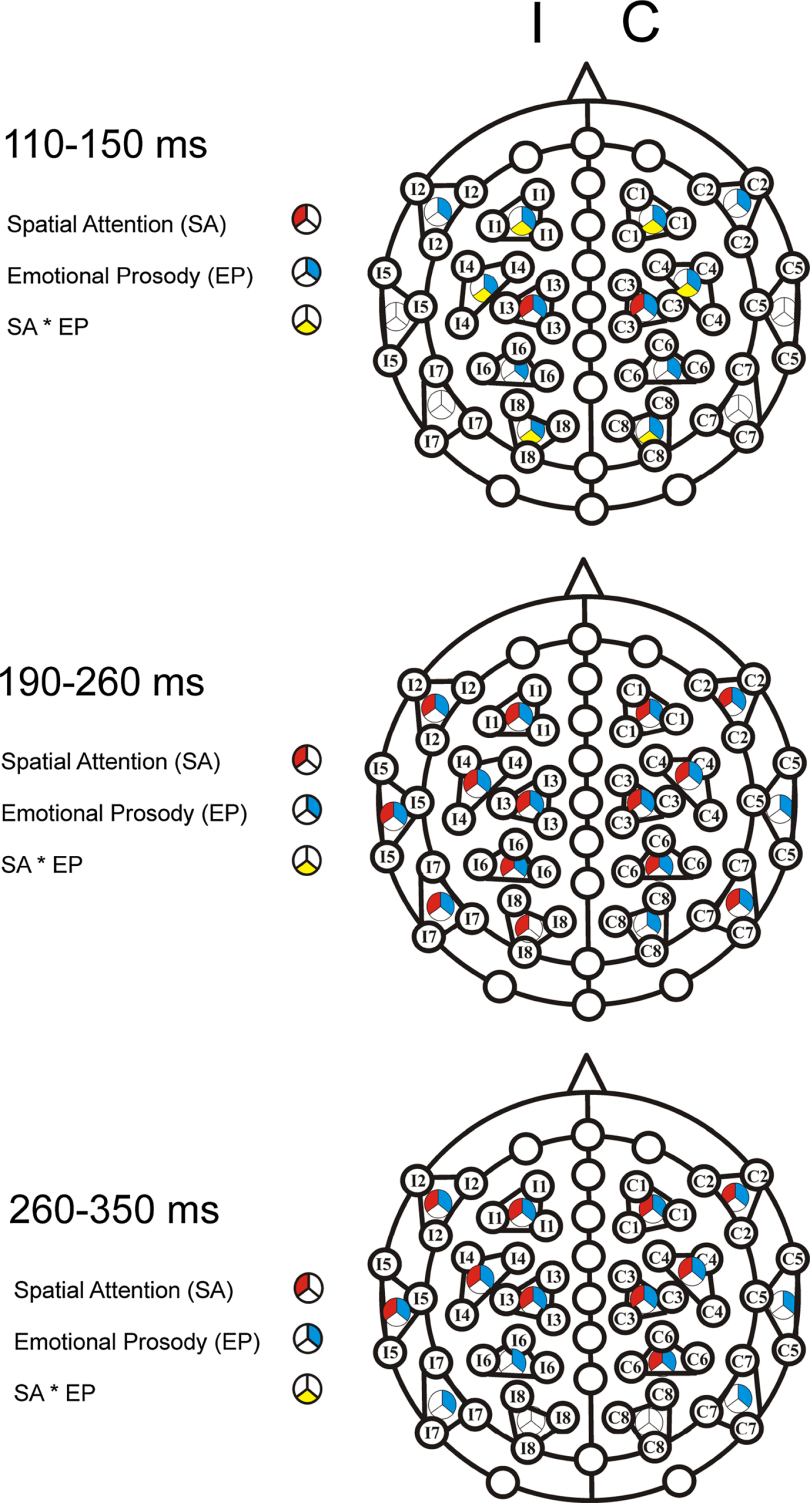
**Schematic drawing of the electrode position and clustering of the electrodes.** Statistical results for single clusters are colour coded: Main effect of Spatial Attention (SA): red, main effect of Emotional Prosody (EP): blue, and the interaction of Spatial Attention and Emotional Prosody (SA * EP): yellow (I = ipsilateral; C = contralateral). Statistical results are illustrated separately for three different time epochs.

Artifacts due to eye movements (difference of two sample points within a segment of the vertical or horizontal EOG or M_1 of larger than 120 μV), muscle activity (channels with voltage differences of more than 160 μV between two adjacent sample points) or amplifier saturation (maximal voltage difference less than 0.5 μV over a time epoch of at least 100 ms) were eliminated prior to averaging. The averaged ERP to standard stimuli of each participant is based on 1898 to 2845 trials (224 – 365 for each of the eight conditions: two attention conditions (attend left vs. right speaker) and four emotional expression conditions). Participants with a rejection rate of higher than 40% of the epochs were discarded. Adjacent recording sites were combined to clusters of three electrodes each (see Figure 1), resulting in eight clusters for each hemisphere. The number of electrode sites (61 sites) was reduced to 16 electrode clusters in order to reduce the number of statistical comparisons and still guarantee a satisfying coverage of the frontal, temporal and parietal scalp of both hemispheres. Each cluster score represents the mean amplitude of three adjacent electrodes. They were located either on the left or right hemisphere of the head.

Since no middle-line electrode was included in the electrode clusters, M_4 was additionally analyzed because it is well known that the auditory vertex potential has its maximal amplitude at this site. Clusters of the left and right hemisphere were converted to the hemisphere ipsilateral or contralateral to the stimulation. This calculation (contralateral versus ipsilateral) was included because we expected the Attention and Emotion effects to be higher at contra- than ipsilateral electrode sites. We report results of the central electrode M_4 first and then report the cluster analysis in order to show the robustness of our results. We report significant contrasts between ERPs following stimuli in different emotional prosodies in Table 4.

**Table 4:** Significant contrasts (post-hoc t-tests, p < .05) between ERPs following stimuli in different emotional prosodies in the three time-windows (PA: position attended, PU: position unattended; I: ipsilateral to stimulation, C: contralateral to stimulation)

**Comparison**	**Superordinate ANOVA**	**110–150 ms**	**190–260 ms**	**260–350 ms**
**neutral – happy**	Significant over both hemispheres	**1,2,3,4,6**	**1,2,3,4,5,6**	**1,2,3,4,5,6,7**
	ipsilateral (I) / contralateral (C) effect			
	position attended (PA) / unattended (PU)	**7 (PU)**		
**neutral – threatening**	Significant over both hemispheres		**1,2,3,4,5,6**	
	ipsilateral (I) / contralateral (C) effect			
	position attended (PA) /unattended (PU)	**7 (PU)**		
**neutral-fearful**	Significant over both hemispheres	**3,6**	**1,2,3,4,6,7**	**1,3**
	ipsilateral (I) / contralateral (C) effect			
	position attended (PA) / unattended (PU)	**4,7 (PU)**		
**happy-threatening**	Significant over both hemispheres	**1,2,3,4,6**	**5, 7**	**1, 2, 3, 4, 5, 6**
	ipsilateral (I) / contralateral (C) effect			
	ipsilateral (I) / controlateral (C) effect		**C3, C6**	
**happy-fearful**	Significant over both hemispheres	**1,2,3,4,6**	**1,2,3,4,5,6,7,8**	**1,2,3,4,6,7**
	ipsilateral (I) / contralateral (C) effect	**C4 (PU)**		
	position attended (PA) / unattended (PU)	**4 (PA)**		
**threatening - fearful**	Significant over both hemispheres	**2,3,6**	**1,2,3,4,5,6,7,8**	**1, 2, 3, 4, 6, 7**
	ipsilateral (I) / contralateral (C) effect	**1,4 (PA)**		
	position attended (PA) / unattended (PU)			

Numbers without the letters I or C denote bilateral cluster activation.

For statistical analyses, mean amplitudes were calculated for the following three time epochs: first time window (110–150 ms), second time window (190–260 ms), and third time window (260–350 ms). Time windows were chosen by visual inspection and mean amplitudes were calculated around peak values of the grand average (N1, P2). We expected early attention and affective prosody effects in the time range of the N1 and P2.

For each time epoch mean amplitudes of ERPs were submitted to ANOVAs comprising the four repeated measurement factors, Spatial Attention (two levels: attended vs. unattended), Emotional Prosody (four levels: neutral, happy, threatening, fearful), Cluster (eight levels), and Hemisphere (two levels: ipsilateral vs. contralateral). Higher order interactions were followed up with appropriate sub-ANOVAs or t-tests. Recordings from the fronto-central electrode M_4 were analyzed without the factors Hemisphere and Cluster.

Additionally, we calculated ERP difference waves (attended minus unattended) for each emotion (neutral, happy, threatening, fearful). An ANOVA including the factors Emotional Prosody (neutral, happy, threatening, fearful), Hemisphere (contra versus ipsilateral) and Cluster (1–8) was run. The ANOVA for the electrode M_4 was run with the factor Emotional Prosody only.

All statistics were computed with the program package SPSS, subroutine GLM for repeated measurements. Greenhouse Geisser -corrected *p-*values are reported [32]. In order to prevent an inflation of the alpha error, the Bonferroni-correction (corrected for six tests comparing the four emotional conditions) was applied.

In the following result section, we first report the behavioral data including d’ scores and IE scores. In the ERP result section we first report the results for site M_4 followed by the results for the analyses including all clusters.

## Results

### Behavioral Data

#### d’ scores

**Figure 2:**
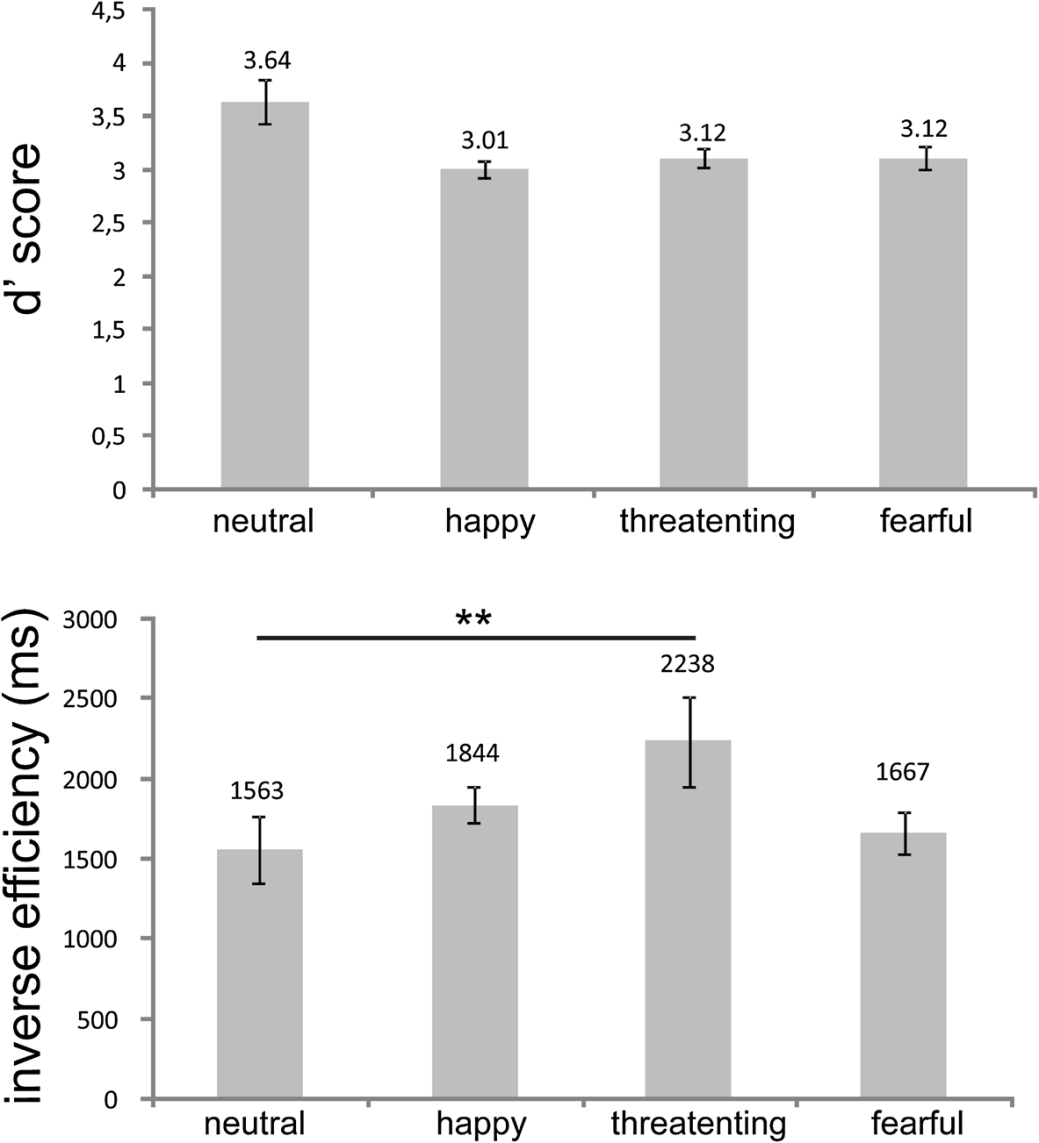
**Mean d’ scores and inverse efficiency scores (ms) with error bars (standard error of the mean) for neutral, happy, threatening and fearful voices.** The horizontal line indicates significant differences between the conditions (* = p < .05, ** = p < .001).

Participants were well able to discriminate the two positions (mean d’ = 3.12, *SE* = .09). Moreover, the ANOVA with the repeated measurement factor Emotional Prosody and d’ scores as dependent variable revealed a main effect of Emotional Prosody (*F*(3,36) = 6.00, *p* < .01). D’ scores tended to be higher for neutral (mean neutral: 3.64, *SE*: 0.21) compared to happy, threatening and fearful voices, although this difference failed to reach significance (mean neutral: 3.64, *SE*: 0.21; mean happy: 3.01, *SE*: 0.08, mean threatening: 3.12, *SE*: 0.09, mean fearful: 3.12, *SE*: 0.11; neutral versus happy: *t*(12) = 2.83, *p* < .1; neutral versus threatening: *t*(12) = 3.01, *p* < .1; neutral versus fearful: *t*(12) = 2.67, *p* > .1) . There were no differences in d’ scores between happy, threatening and fearful emotional prosodies (all *p*s > .1) (see Figure 2).

### IE scores (ms)

Using IE scores as dependent variable, the main effect of Emotional Prosody was marginally significant (*F*(3,36) = 3.49, *p* < .1). IE scores were lower for neutral (mean: 1563 ms, *SE* = 204) compared to threatening voices (mean: 2238 ms, *SE* = 283) (*t*(12) = −4.906, *p* < .01 (see Figure 2)). All the other comparisons were not significant (*p* > .1).

### Event-related brain potentials

#### M_4

**Figure 3:**
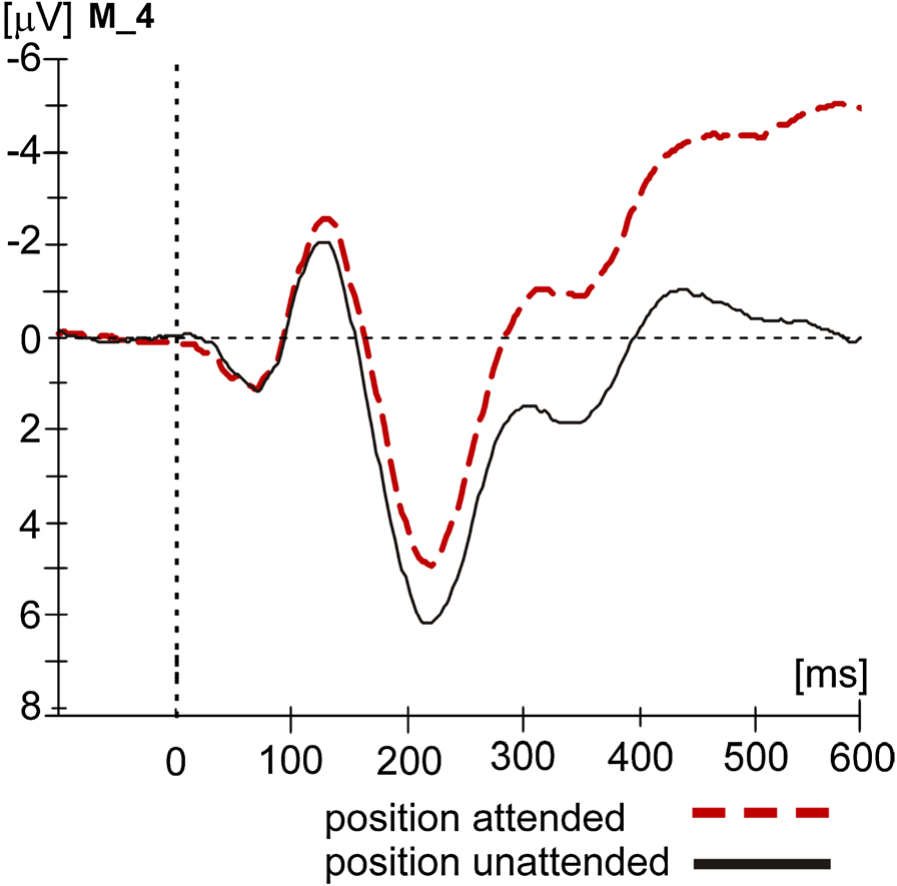
ERPs to standard stimuli when spatially attended (red dashed line) vs. when spatially unattended (black solid line), recorded at the central electrode M_4 (positioned between Fz and Cz of the international 10–20 system).

**Figure 4:**
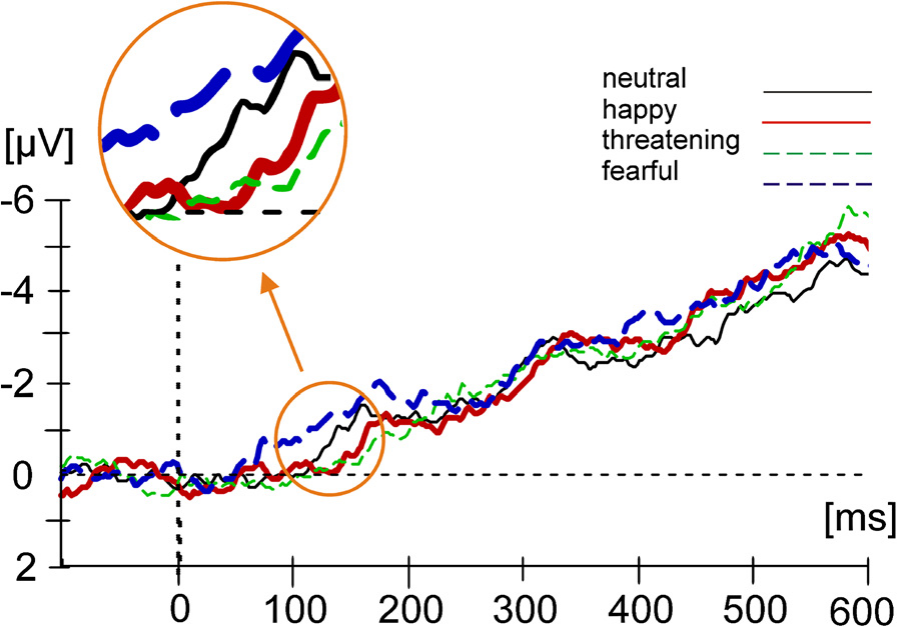
ERP difference waves: ERPs to attended stimuli minus ERP to unattended stimuli separately for the four emotional prosodies, recorded at the central electrode M_4 (positioned between Fz and Cz of the international 10–20 system).

**Figure 5:**
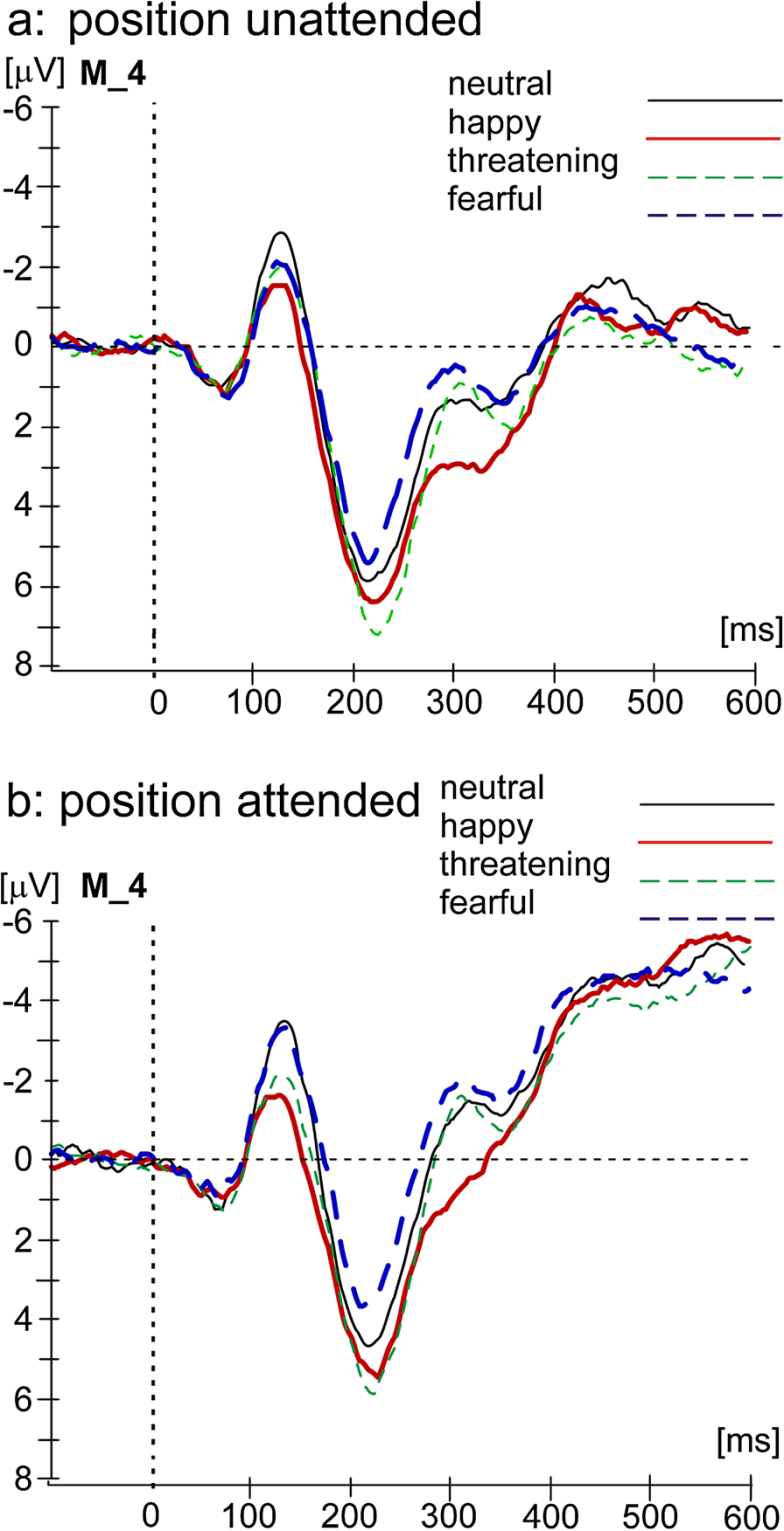
ERPs to standard stimuli in different emotional prosodies when spatially unattended (a) and when spatially attended (b) recorded at the central electrode M_4 (positioned between Fz and Cz of the international 10–20 system).

See Figure 3 for the spatial attention effect, Figure 4 for difference waves (ERP (attended) minus ERP (unattended)), and Figure 5 for the Emotional Prosody effect at the attended and unattended position for recording site M_4.

110–150 ms: In the latency range of the N1 the overall ANOVA revealed a main effect of Spatial Attention (*F*(1, 12) = 10.16, *p* < .01), a main effect of Emotional Prosody (*F*(3, 36) = 29.81, *p* < .001), and an interaction of Spatial Attention and Emotional Prosody (*F*(3, 36) = 3.50, *p <* .05). The N1 was more negative going to spatially attended than spatially unattended standards (Figure 3, difference waves in Figure 4). Separate subordinated ANOVAs for different emotional prosodies found a significant enhancement of the N1 to standard stimuli due to Spatial Attention for fearful stimuli (fearful stimuli: main effect Spatial Attention: *F*(1,12) = 19.92, *p* < .01), while this effect was not significant for neutral, happy, and threatening stimuli (neutral: *F*(1,12) = 2.84, *p* > .1*;* happy: *F*(1,12) = .65, *p* > .1; threatening: *F*(1,12) = .20, *p* > .1).

Moreover, subordinated ANOVAs confirmed that the effect of Emotional Prosody was significant at both the attended and the unattended location but the higher F-value for the attended (position attended: *F*(3,36) = 43.89, *p* < .001) than the unattended position (position unattended: *F*(3,36) = 5.55, *p* < .01) suggests a stronger Emotional Prosody effect at the attended location (Figure 5). Post-hoc t-tests revealed that ERPs to happy stimuli were significantly less negative going than ERPs to neutral stimuli at both attended and unattended positions (neutral vs. happy: attended: *t*(12) = −8.57, *p* < .01; unattended: *t*(12) = −3.78, *p* < .05). The ERP to threatening stimuli was less negative going than the ERP to neutral stimuli only if stimuli were attended (neutral vs. threatening: attended: *t*(12) = −5.50, *p* < .01; unattended *t*(12) = −2.33, *p* > .1). The ERP to fearful stimuli did not differ from the ERP to neutral stimuli, but the ERP to fearful stimuli was significantly more negative than the ERP to happy stimuli at the attended position (happy vs. fearful: attended: *t*(12) = 12.03, *p* < .01; unattended: *t*(12) = 2.71, *p* > .1)*.* At the attended position ERP differences between threatening and fearful stimuli reached the significance level (*t*(12) = 8.47, *p* < .01) while the difference between happy and threatening voices was only marginally significant (happy vs. threatening: *t*(12) = 3.12, *p* < .1). Happy stimuli elicited the least negative going ERP, followed by threatening stimuli.

The ANOVA for the difference waves (ERP (attended) minus ERP (unattended), see Figure 4) found a significant main effect of Emotional Prosody (*F*(3, 36) = 3.50, *p* < .05)^b^.

190–260: The overall ANOVA revealed a highly significant main effect of Spatial Attention (*F*(1,12) = 13.40; *p* < .01) in the absence of a significant interaction of Spatial Attention and Emotional Prosody (*F*(3,36) =.37, *p* > .1). The Spatial Attention effect consisted of a more negative ERP to stimuli at attended positions (see Figures 3 and 4).

In addition, the overall ANOVA found a significant main effect of Emotional Prosody (*F*(3,36) = 30.85, *p* < .001). Post-hoc t-tests comparing the different emotion conditions averaged across the two levels of Spatial Attention revealed that the ERP to neutral stimuli was significantly different from ERPs to stimuli in all other emotional prosodies (neutral vs. happy: *t*(12) = −3.39, *p* < .05; neutral vs. threatening: *t*(12) = −3.90, *p* < .05; neutral vs. fearful: *t*(12) = 4.21, *p* < .01*):* Fearful stimuli elicited a more negative ERP than neutral stimuli, and happy and threatening stimuli elicited less negative going ERPs than neutral stimuli. The differences between ERPs to happy and fearful stimuli and between threatening and fearful stimuli were significant, too (happy vs. fearful: *t*(12) = 8.54, *p* < .01; vs. fearful: *t*(12) = 7.11, *p* < .01*,* see Figure 5).

**Figure 6:**
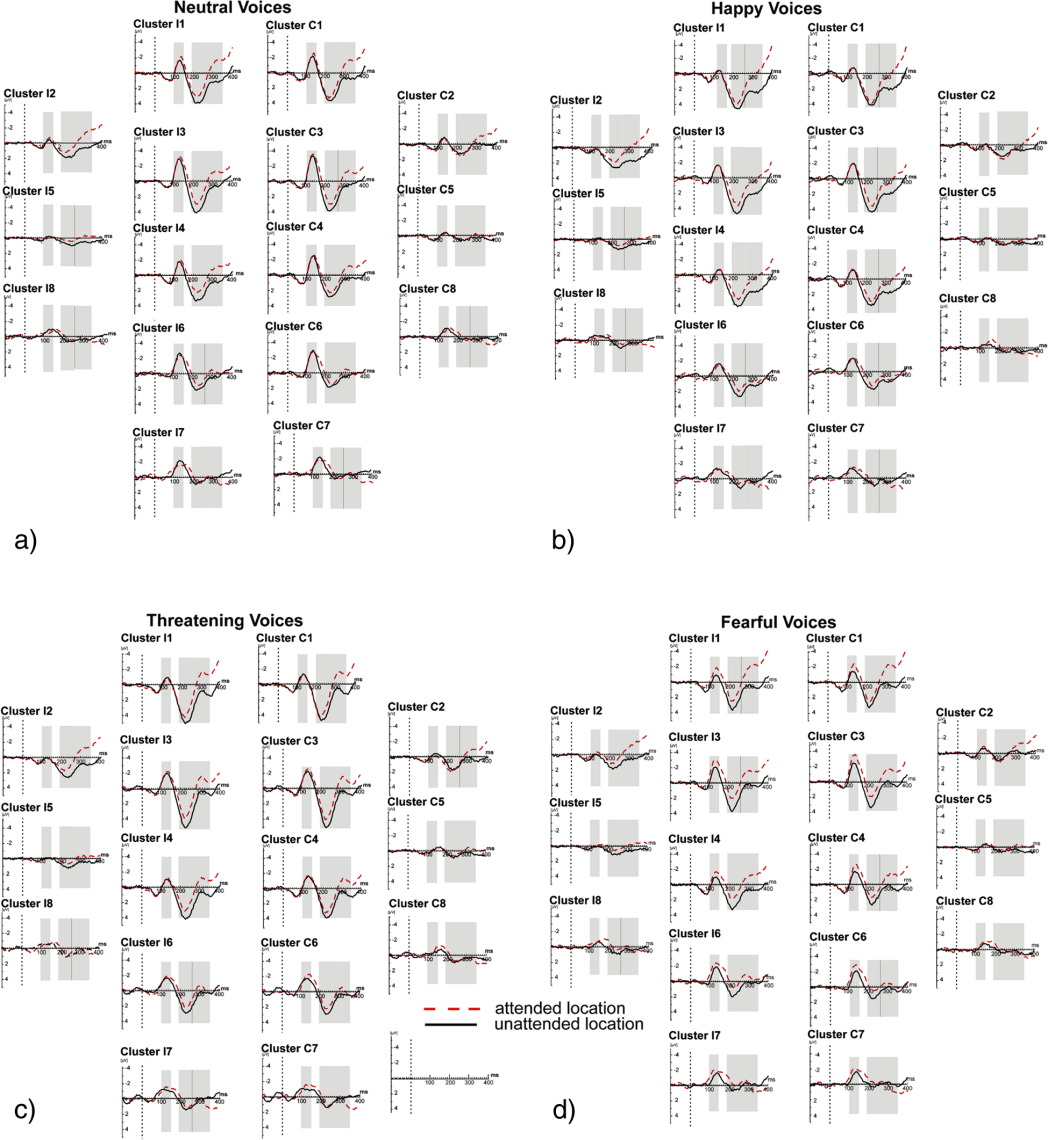
**a-d. Grand mean event-related potentials to voice stimuli with a) a neutral prosody, b) a happy prosody, c) a threatening prosody and d) a fearful prosody.** Red dashed line: ERPs to attended locations, black solid line: ERPs to unattended locations. Grey shaded areas indicate the time epochs used for the statistical analyses.

The ANOVA for the difference waves (attended minus unattended) did not reveal a main effect of Emotional Prosody (*F*(3,36) = .37, *p* > .1, see also Figure 4 and endnote^b^).

260–350: The overall ANOVA revealed a highly significant main effect of Spatial Attention (*F*(1,12) = 36.18, *p* < .001) but no interaction of Spatial Attention and Emotional Prosody (*F*(3,36) = .07, *p* > .1). The Spatial Attention effect consisted of a more negative ERP to stimuli at attended positions (see Figures 3 and 4). The overall ANOVA found a significant main effect of Emotional Prosody (*F*(3,36) = 28.97, *p* < .001, see Figure 5). As revealed by post-hoc t-tests, the ERP to happy stimuli was less negative than the ERP to neutral voices (neutral vs. happy: *t*(12) = −5.82, *p* < .01), whereas the ERP to fearful stimuli was only marginally significant more negative than the ERP to neutral stimuli (neutral vs. fearful: *t*(12) = 2.80, *p* < .1). The ERP to threatening stimuli did not differ significantly from the ERP to neutral stimuli, but ERP-differences between happy and threatening (*t*(12) = 4.97, *p* < .01) as well as between threatening and fearful stimuli (*t*(12) = 3.97, *p* < .05) reached the significance level. Moreover, ERPs to happy and fearful stimuli differed from each other (happy vs. fearful: *t*(12) = 9.95, *p* < .01).

The ANOVA for the difference waves (attended minus unattended) did not reveal an effect of Emotional Prosody (*F*(3,36) = .07, *p* > .1, see Figure 4 and endnote^b^).

### Cluster

Figure 6 shows the grand mean event-related potentials to voice stimuli with a neutral (a), a happy (b), a threatening (c) and a fearful (d) prosody.

Figure 1 (see methods section) summarizes the corresponding significant main effects of Spatial Attention and Emotional Prosody, and the interactions of these factors for the three time windows and for each cluster.

110–150 ms: In the overall ANOVA the interactions of Spatial Attention and Emotional Prosody (*F*(3,36) = 3.58, *p* < .05), of Spatial Attention and Cluster (*F*(7,84) = 3.51, *p* < .05) as well as of Spatial Attention, Emotional Prosody, and Cluster reached significance (*F*(21, 252) = 3.12, *p* < .05). The Spatial Attention effect was significant at cluster 3 (*F*(1, 12) = 7.18, *p* < .05), and marginally significant at clusters 1 and 4 (*p*s < .1). When analyzing emotional prosodies separately, a main effect of Spatial Attention was found for fearful stimuli (ANOVA for fearful stimuli only: main effect of Spatial Attention: *F*(1, 12) = 17.10; *p* < .01; interaction of Spatial Attention and Cluster: *F*(7, 84) = 7.41, *p <* .01); this effect was reliable at clusters 1, 3, 4, 6, and 7 (*p*s < .05)). An interaction of Spatial Attention and Cluster was significant for threatening stimuli (*F*(7, 84) = 3.35; *p* < .05) as well. However, no more than a marginally significant main effect of Spatial Attention was found for Cluster 7 (*F*(1, 12) = 3.62; *p* < .1).

Moreover, the overall ANOVA revealed a highly significant main effect of Emotional Prosody (*F*(3,36) = 27.72, *p* < .001), and an interaction of Emotional Prosody and Cluster (*F*(21,252) = 9.91, *p* < .001). Main effects of Emotional Prosody were highly significant at the attended position (*F*(3, 36) = 18.24, *p* < .001) as well as at the unattended position (*F*(3,36) = 12.22, *p* < .001). The Emotional Prosody * Cluster interaction was significant at both the attended and the unattended position (*p*s < .05). At clusters 1, 2, 3, 4, and 6, main effects of Emotional Prosody were confirmed for stimuli at the attended (all *p*s < .01) as well as for stimuli at the unattended position (all *p*s < .01). At the posterior clusters 7 and 8 the Emotional Prosody effect reached significance only at the unattended position (*p*s < .05). Table 4 summarizes the significant comparisons between emotional prosodies (posthoc t-tests) at the different clusters.

The ANOVA for the difference waves (attended minus unattended) revealed a significant effect of Emotional Prosody (*F*(3,36) = 3.58, *p* < .05; see endnote^b^).

190–260 ms: More negative ERPs in the attended than in the unattended condition were observed. Because of significant interactions of Spatial Attention and Cluster (*F*(7,84) = 11.42, *p* < .01) as well as of Spatial Attention, Hemisphere and Cluster (*F*(7,84) = 6.53, *p* < .05) in the overall ANOVA, separate ANOVAs for single clusters were calculated (see Figure 1 (methods section)). The Spatial Attention effect was significant for all clusters (all *p*s < .05), and with the exception of cluster 7, the interaction of Spatial Attention and Hemisphere was significant for all clusters (all *p*s < .05) as well. At clusters 1, 3, 4, and 6, the main effect of Spatial Attention was found at contralateral as well as at ipsilateral clusters (all *p*s < .05). At clusters 2, 5, and 8, this effect was significant only at ipsilateral clusters (all *p*s < .05). Moreover, the overall ANOVA revealed a highly significant main effect of Emotional Prosody (*F*(3,36) = 23.44, *p* < .001), and a significant interaction of Emotional Prosody and Cluster (*F*(21, 252) = 14.16, *p* < .001). A significant main effect of Emotional Prosody was found at all 8 clusters (all *p*s < .05). Table 4 summarizes the significant comparisons between emotional prosodies (posthoc t-tests) at the different clusters.

The ANOVA for the difference waves (attended minus unattended) did not reveal a significant main effect of Emotional Prosody (*F*(3,36) = .432, *p* > .1; see endnote^b^).

260–350 ms: A significant main effect of Spatial Attention was observed (*F*(1,12) = 7.99, *p* < .05). The interactions of Spatial Attention and Cluster (*F*(7,84) = 23.41, *p* < .001), of Spatial Attention and Hemisphere (*F*(1,12) = 11.12, *p* < .01), and of Spatial Attention, Hemisphere, and Cluster (*F*(7,84) = 4.85, *p<* .05) reached the significance level as well. The Spatial Attention effect was reliable at clusters 1 to 5 (all *p*s < .01) (Figure 1 in the methods section). At these clusters and at cluster 6, the Spatial Attention * Hemisphere interaction reached significance, (all *p*s < .05). At clusters 1, 3, and 4, the Spatial Attention effect was found at ipsilateral as well as at contralateral clusters (all *p*s < .01). At clusters 2 and 5, the effect was significant only at ipsilateral clusters (*p*s < .01), at contralateral clusters marginal effects were observed (*p*s < .1).

Moreover, in the overall ANOVA the main effect of Emotional Prosody (*F*(3,36) = 21.97, *p* < .001) as well as the Emotional Prosody by Cluster interaction were highly significant (*F*(21,252) = 20.42, *p* < .001). The effect of Emotional Prosody was significant at all clusters (all *p*s < .01) with the exception of the posterior cluster 8.

The ANOVA for the difference waves (attended minus unattended) did not reveal an effect of Emotional Prosody (*F*(3,36) = .22, *p* > .1; see also endnote^b^).

In sum, the posthoc t-tests for single clusters largely confirmed the results obtained for M_4 (see Table 4 for a summary).

## Discussion

The present study investigated effects of spatial attention and emotional prosody on the processing of vocal stimuli. Two-syllable pseudo-words spoken in four emotional prosodies (neutral, happy, threatening, and fearful) were presented in a random order from two spatial positions. Participants attended to one position only in order to detect infrequent deviant stimuli. Both behavioral and ERP indices of stimulus processing were assessed. The main findings were as follows:

Even though marginally significant, neutral targets were detected with a higher precision than targets spoken in a happy, threatening or fearful prosody.

ERPs differed as a function of emotional prosody both at the spatially attended and spatially unattended location. Importantly, the early spatial attention effect (N1) was mostly pronounced for fearful stimuli. The N1 following fearful stimuli was more negative than the ERPs following neutral stimuli, while ERPs elicited by happy and threatening stimuli were less negative than ERPs to neutral stimuli. The Emotional Prosody effect was significant for stimuli at attended and unattended positions.

### Behavioral processing of vocal prosody

In most previous studies, aversive stimuli have been found to be detected faster than neutral or positive emotional stimuli [33-36]. By contrast, in the present study, detection rates were higher and processing was more efficient for neutral compared to happy, threatening and fearful stimuli. In the present study targets were defined as two different rather than two identical syllables. If the emotional prosody is partially automatically extracted, as suggested by the present results, it seems plausible to assume that the emotional tone might have distracted the participants and thereby caused lower target identification based on syllables. However, this speculation has to be treated with caution since the number of targets was relatively low.

### Early ERP modulations by spatial attention for fearful human voices

The finding of an enhanced negativity starting in the latency range of the N1 replicates once again the well known spatial attention effect discovered by Hillyard et al. [37]. Early attention effects starting around 100 ms post-stimulus are generally found for easy to discriminate channels such as two locations one in the left and one in the right hemifield [38].

However, it might be wondered why reliable spatial attention effects starting in the N1 time range were mainly seen for fearful voices while later ERP spatial attention effects emerged similarly for all emotional prosodies. A specific processing for fearful stimuli has often been reported [39-41] and has been seen as adaptive in a social context.

On the other side, it has to be noticed that relatively long ISIs were used in the present study. It is well known that N1 attention effects are most likely if short ISIs are employed [42]. This might explain why attention effects for most of the emotional voices were observed relatively late. However, on this background, the earlier emergence of spatial attention effects for fearful stimuli stress the preferred processing and specific enhancement of the processing of these stimuli by spatial attention.

According to Treue [43], bottom-up features of the stimulus itself and attentional top-down influences are integrated in a common saliency map which is a representation of the environment that weighs every input by its sensory features and behavioral relevance. Stimuli of high salience are processed even if unattended, while the processing of less salient unattended stimuli is suppressed. Applied to the present study, the spatial attention effect in the N1 time range might be interpreted as a release of an active suppression of the processing of task irrelevant fearful stimuli or as a further enhancing of the processing of this stimulus class. The finding that the N1 amplitude of ERPs to fearful stimuli arose as a function of spatial attention to the level of the N1 to neutral stimuli might be interpreted as evidence for the first interpretation.

It might be argued that emotion specific features (such as duration and low level acoustic characteristics) cause the differences between ERPs to emotional stimuli.

Of course, we cannot rule out the possibility that other low-level features contribute to the N1 Emotional Prosody effects. Accordingly, previous research has suggested that a number of acoustic features such as fundamental frequency (f0) and intensity differ among different emotional utterances [44,45]. As described, our stimuli varied in fundamental frequency as a function of emotional category as well, suggesting that our actors were able to produce valid stimuli. Eliminating these features would be equal to eliminating emotional prosody. However, there are many arguments, why our results cannot be fully accounted by simple differences in physical stimulus features: First, we controlled for the duration of stimuli across all emotional prosodies. Second, the successful control of physical stimulus features is supported by similar latencies of the vertex potential across the four emotional prosodies (see Figure 5). Third, physical stimulus features would be expected to mainly affect exogenous ERPs, in other words maximally the vertex potential. However, we observed ERP differences as a function of Emotional Prosody for all analyzed time epochs. Fourth, ERP differences due to physical stimulus features are supposed to be independent of top-down modulation that is attention in our study. However, The N1 amplitude differences between different emotional prosodies were different for the attended and the unattended condition.

### Modality specific processing of emotions and the influence of spatial attention

The present results coincide with the results reported by Sauter and Eimer [6] who used human vocalizations as stimuli and found an ERP positivity (150–180 ms) for different emotions (fear, disgust, achievement) compared with their spectrally rotated counterparts analog to previous findings for emotional faces [39] and pictures [46].

Similar findings for the processing of auditory and visual affective stimuli are surprising, however. For auditory processing, the existence of subcortical pathways to the amygdala has been demonstrated by studies of fear conditioning to acoustic stimuli in rats and guinea pigs [47,48]. This subcortical route has been suggested to be particularly fast and to enable a quick detection of emotionally relevant stimuli. For the processing of facial stimuli, several studies have proposed a fast subcortical pathway as well [49-51]. However, according to Pessoa and Ungerleider [52] such a short route has not yet been unequivocally demonstrated in the visual system. Indeed, Pessoa and Ungerleider [52] have found an enhanced processing of emotional faces only when they were attended. These authors argue that a detailed analysis of facial features is rather impossible via a subcortical route (see [52] for a detailed discussion). This would argue for a quicker and more automatic processing of auditory emotional stimuli compared to visual emotional stimuli. On the other hand, the identification of the valence of auditory stimuli requires the integration of the auditory stream across an extended time epoch while visual scenes are picked up at one glance. However, in contrast to visual stimuli which in most cases need to be foveated in order to be identified, no overt shifts of attention are necessary to identify auditory stimuli. In line with this reasoning we found that ERPs differed as a function of emotional prosody both in the spatially attended and the spatially unattended channel. Thus, the emotional valence of auditory stimuli might be partially extracted automatically or at least without spatial attention. This conclusion is in agreement with the brainimaging results of Klinge et al. (2010) [53] who did not find a difference in amydala activation as a function of whether or not the emotional prosody of voices had to be attended. By contrast another fMRI study found that when all auditory input had to be completely discounted (intermodal attention) [26] a differential activation of the amydala for different emotional prosodies did not emerge. By manipulating spatial attention within the auditory modality, our experiment more resembles the within modality manipulation of Klinge et al. (2010) [53].

Therefore, we interpret our results in line with reports suggesting that the emotional valence of stimuli can be extracted in the absence of (at least spatial) attention, but that attention nevertheless modulates emotional processing [23,54].

Finally, it has to be pointed out that we observed different prosody effects for early and later ERPs, i.e. processing steps. This observation might be related to the two roads of emotion processing proposed by LeDoux (cited from [55]). The “quick but dirty low road” is assumed to mediate an automatic processing of both attended and unattended events. While it seems not to be able to fully distinguish the whole range of emotional prosodies, e.g. two different aversive stimulus classes, such as fearful and threatening stimuli, at least not at unattended locations, the slower “high road” allows for a more elaborated and differential processing of both attended and unattended stimuli. This finding would suggest that later ERP effects of emotional prosody are independent of spatial attention.

## Conclusion

The present results suggest that while emotional prosody is processed independent of spatial attention, spatial attention nevertheless modulates the degree of voice processing as a function of emotional valence at sensory processing stages. By contrast, at later stages emotional prosody is processed independent of the focus of spatial attention. Further research has to investigate whether this rule holds for stimuli of other modalities or other types of attention (such as intermodal attention, conscious vs. unconscious processing) as well.

## Endnotes

^a^Originally, the main experiment comprised an additional orthogonally manipulated factor. Participants had to selectively attend to one voice only. However, the voices of the two female speakers were too similar and participants did not manage to distinguish between them. Even after excluding participants (n = 4) with very low performance in discriminating the voices (d’ < 0.4), mean d’ (calculated as *d’ = z(p(hit)) - z(p(FA*)) (FA = False Alarms) for the remaining participants (n = 13) was low (d’ = .67; SE = .08).

^b^See results of the overall ANOVA (The main effect Emotional Prosody in the ANOVA of the difference waves corresponds to the Spatial Attention * Emotional Prosody interaction in the overall ANOVA).

## Abbreviations

EEG: Electro-encephalogram; ERP: Event-related potential; STS: Superior temporal sulcus; EOG: Electro-occulogram; FA: False alarm; RT: Reaction times; IE score: Inverse Efficiency score; ANOVA: Analysis of Variance.

## Authors’ contributions

JG and BR designed the experiment. JG run the ERP experiment. JG, JF and BR analyzed the data. JG, JF, and BR wrote the paper. All authors read and approved the final manuscript.
